# Use of Rituximab in Autoimmune Hemolytic Anemia Associated with Non-Hodgkin Lymphomas

**DOI:** 10.1155/2011/960137

**Published:** 2011-04-10

**Authors:** Claudio Fozza, Maurizio Longinotti

**Affiliations:** Institute of Hematology, University of Sassari, Viale San Pietro 12, 07100 Sassari, Italy

## Abstract

The association between non-Hodgkin lymphomas and autoimmune disorders is a well-known event. Also autoimmune hemolytic anemia (AHA), although much more frequent in patients with chronic lymphocytic leukemia (CLL), has been described in this group of patients. In recent years, among the more traditional therapeutic options, rituximab, an anti-CD20 monoclonal antibody, has shown interesting results in the treatment of primary AHA. Although this drug has been frequently used for AHA in patients with CLL, much less data are available on its use in NHL patients. However, considering that the main pathogenetic mechanism of AHA in course of lymphoproliferative disorders seems to be an antibody production directly or indirectly mediated by the neoplastic clone, this monoclonal antibody represents an ideal therapeutic approach. In this paper we will briefly describe some biological and clinical features of NHL-patients with AHA. We will then analyze some studies focusing on rituximab in primary AHA, finally reviewing the available literature on the use of this drug in NHL related AHA.

## 1. Autoimmune Hemolytic Anemia in Non-Hodgkin Lymphomas

The association between lymphomagenesis and autoimmunity is a well-known scenario mainly derived from reports describing both patients and mouse strains in which autoimmune manifestations and lymphoproliferative disorders coexisted [1]. On the other hand in recent years population-based studies have specifically shown that several autoimmune disorders are associated with an increased risk in developing non-Hodgkin lymphomas (NHL) [2, 3]. Some factors have been specifically advocated as possible explanations for this association, such as chronic immune stimulation, immunomodulating drugs, and common genetic or environmental variables. Lymphomagenesis and autoimmunity could be pathogenetically put together by considering that both of them result from the impairment of the physiologic mechanisms which should control lymphoid cell growth and differentiation [4]. 

Autoimmune hemolytic anemia (AHA), although much more frequent in patients with chronic lymphocytic leukemia [5], has been described also in a small proportion of NHL, patients, ranging from 3 to 6% in different cohorts [6, 7]. Besides several anecdotal reports [8], clinical and prognostic features of patients with AHA have been investigated in a large cohort of NHL patients. In this study 16 subjects (3%) with AHA have been selected among 517 NHL patients. Ten patients had B-cell NHL, 5 T-cell NHL and 1 poorly differentiated NHL while follicular lymphoma was the most represented subtype, having being diagnosed in 5 subjects. While looking at different subtypes of AHA warm and cold antibodies were identified in 13 and 3 patients respectively. Patients with AHA were more likely to have T-cell NHL when compared to the whole cohort (33% versus 14%). A significantly higher proportion of patients with NHL-associated AHA were diagnosed with monoclonal gammopathy (8% versus 25%). Moreover patients with AHA showed a trend towards a worse response rate to antilymphoma treatments and were apparently characterized by a reduced overall survival, although one may speculate that this finding represents just a surrogate of the worse prognosis correlated to both T-cell phenotype and paraproteinemia. Finally, in most patients a discrepancy among time to response of NHL and AHA was observed, thus suggesting possible different pathways mediating response to treatment [6]. 

In another series evaluating 370 NHL patients AHA was detected in 23 patients (6.2%), 19 with warm-type and 4 with cold-type antibodies. This report then focused on the 4 cases with cold agglutinin disease (CAD), showing then that all of them were having low-grade NHL and had monoclonal IgM gammopathy. In 3 of them both NHL and AHA showed a good response to chlorambucil plus prednisone [7].

A somehow specular point of view has been recently offered by the above-mentioned case population study on patients with autoimmune manifestations, which showed that even AHA, although much less than other autoimmune diseases, is associated with a 2.6-fold increase in NHL risk. This association is even more evident among subjects with long AHA duration and is stronger for B-cell NHL, especially for the diffuse large B-cell lymphoma subtype [2]. Another report has also suggested that AHA could be preferentially associated with other NHL subtypes, such as marginal zone NHL and T-cell lymphomas [3].

## 2. Rituximab in Primary Autoimmune Hemolytic Anemia

In recent years, among the more traditional therapeutic options, such as steroids and other immunomodulating drugs, also monoclonal antibodies have become available for the treatment of AHA. Apart from limited experiences with alemtuzumab [9], rituximab has shown the most interesting results. This monoclonal antibody is directed against the CD20 antigen expressed on B-lymphocytes and is widely used in the treatment of lymphoproliferative disorders. More in details, it has been nowadays incorporated in the first line treatment of both indolent [10] and aggressive NHL [11], having offered in both conditions a marked improvement in terms of response rates and overall survival. 

In the preliminary studies on the use of rituximab on AHA, after the first report on a child with AHA associated to pure red cell aplasia successfully treated with rituximab and intravenous immunoglobulins [12], 13 out of 15 children with warm antibody AHA showed responses to rituximab [13]. On the other hand, it was also highlighted as in 4 patients with Evan's syndrome either immune thrombocytopenia or AHA only responded, but not both [14]. 

Besides several case reports, in recent years three manuscripts have focused on larger patient cohorts. D'Arena and colleagues described the use of rituximab in warm-type idiopathic AHA. All patients were refractory to steroids and/or immunosuppressive drugs and all were given weekly rituximab 375 mg/m^2^ for four consecutive weeks. An increase in hemoglobin levels was observed in all cases, with a mean increment of 3.3 g/dL. At a mean followup of 604 days, 8 patients were still in complete remission and 3 in partial remission [15]. Recently, the effects of 68 courses of rituximab in 53 Belgian patients with AHA were reported. All patients were given rituximab after failing at least one previous line of treatment, including splenectomy in 19% of them. Overall response rates were 79%, with a median followup since first rituximab administration of 15 months. Progression-free survival at 1 and 2 years was 72% and 56% respectively [16]. The efficacy and safety of rituximab was also evaluated in a retrospective study including 27 adults with warm antibody AHA refractory to several previous lines of therapy. Overall, 8 patients achieved a complete response and 17 a partial response. After a mean followup of 21 months, 5 patients relapsed and 3 of them were successfully retreated with rituximab [17].

## 3. Use of Rituximab in Autoimmune Hemolytic Anemia Associated with Non-Hodgkin Lymphomas

The rational of using rituximab in NHL patients experiencing AHA is dual. First of all, as above mentioned, this drug has been demonstrated to play a relevant role in the treatment of primary AHA, having been widely tested in patients refractory to previous line of treatment [12–17]. Moreover, as the main pathogenetic mechanism of NHL related AHA seems to be an antibody production directly or indirectly mediated by the neoplastic clone [18, 19], exploiting a well-demonstrated antilymphoma treatment is undoubtedly an intriguing strategy.

Ten years ago, rituximab has been frequently employed in AHA occurring in chronic lymphocytic leukemia (CLL) [30], and its use has been recently incorporated in the guidelines from the International Workshop on CLL [31]. The experience in using rituximab in NHL patients with AHA is much less robust and so far, besides some reports on patients with concomitant CAD and lymphoproliferative disorders [32–34], ten single cases have been reported between 1998 and 2009 [20–29], as shown in Table 1. The more frequent histological patterns are lymphoplasmacytic [22–24] and marginal zone NHL [20, 25–27, 29], described in 4 and 5 cases respectively. A patient with splenic T-cell angioimmunoblastic NHL has been also described, representing the only reported case in which rituximab was unsuccessful [28]. In 6 out of 10 cases, cold agglutinins were detected while mixed and warm autoantibodies were present in 1 and 3 cases, respectively. All the patients were refractory to at least one previous treatment and 5 out of the 9 patients who then responded to rituximab had previously received three or more lines of treatment. In most cases rituximab was administered at a dose of 375 mg/m^2^ weekly for four weeks, sometimes in association with steroids or other medications. In all the reported case, with the exception of the splenic T-cell angioimmunoblastic NHL, a complete remission of AHA occurred within the first month from the first rituximab administration and responses were usually long lasting. As most of these case reports describe patients with indolent NHL, in which disease eradication is usually not a primary goal of the treatment, the degree of response of the lymphoproliferative disorders to rituximab was usually not reported. Interestingly a patient with small lymphocyte NHL who developed severe hemolytic anaemia due to posttransfusional alloimmunization, was very recently reported to respond to a rituximab-based immunochemotherapy [35]. The efficacy of rituximab was also reported in a case in which both an aggressive form of multicentric Castleman disease and AHA responded [36]. 

As above mentioned, some reports evaluated the efficacy of rituximab in patients with CAD, some of which had concomitant lymphoproliferative disorders. Berentsen and colleagues reported about the use of rituximab in the context of a population-based clinical study of 86 patients with primary chronic CAD, 76% of which had concomitant NHL. Thirty-three patients had lymphoplasmacytic lymphoma, 5 marginal zone lymphoma, 4 small lymphocytic B-cell lymphoma or, CLL and 8 undefined clonal lymphocytosis. In such a context rituximab was the only successful approach, showing response rates around 60%, apparently nondifferent among patients suffering or not from NHL [32]. The same group reported on 37 courses of rituximab monotherapy administered within a prospective trial to 27 patients with primary CAD, 19 of which with concomitant NHL. Fourteen of the 27 patients responded to the first course of rituximab showing 1 complete and 13 partial remissions, according to the response criteria defined in the study. Eight patients relapsed and were retreated with rituximab, in some patients associated with interferon, resulting in 5 partial remissions. On the whole, responses were achieved after 20 of 37 courses of rituximab. Median time to response was 1.5 months, while median increase in hemoglobin level was 4.0 g/L. The median response duration was 11 months and all but one responders observed for more than 12 months relapsed [33]. Slightly lower response rates have been reported in a prospective study of 20 patients with chronic CAD, 7 of which had lymphoproliferative disorders [34]. 

In conclusion, although it should be noted that patients unsuccessfully treated with rituximab are much less likely to be reported, the analysis of the available literature suggests that this monoclonal antibody represents a valid therapeutic option in the treatment of AHA in NHL patients. Keeping in mind that a high proportion of patients relapse after an initial response to rituximab, it remains to be determined if this drug may play a role even in the maintenance. Considering both the pathogenetic mechanisms underlying AHA in NHL patients as well as the poor response rates offered by other therapeutic alternatives, we conclude that rituximab can be considered a valid strategy in NHL patients with steroid refractory AHA as well as a possible option for first-line treatment in selected cases.

## Figures and Tables

**Table 1:** Reported cases of non-Hodgkin lymphoma-associated autoimmune hemolytic anemia treated with rituximab.

First author and year of publication	NHL subtype	Antibodies	Response to rituximab	Previous treatments
Lee and Kueck 1998 [20]	Marginal zone	Cold	Yes	Steroids, cyclophosphamide, plasma exchange
Bauduer 2001 [21]	Lymphoplasmacytic	Cold	Yes	Steroids, cyclophosphamide, cyclosporine, plasma exchange
Cohen et al.2001 [22]	Lymphoplasmacytic	Cold	Yes	Steroids
Layios et al. 2001 [23]	Lymphoplasmacytic	Cold	Yes	Steroids, cyclophosphamide, plasma exchange, vincristine
Mori et al. 2002 [24]	Lymphoplasmacytic	Cold	Yes	Steroids, splenectomy, chop
Paydas et al. 2003 [25]	Marginal zone	Warm	Yes	Steroids
Petit et al. 2003 [26]	Marginal zone	Cold	Yes	Steroids, chlorambucil
Fabbri et al. 2006 [27]	Marginal zone	Warm	Yes	Steroids, splenectomy, cvp
Win et al. 2007 [28]	Splenic T-cell angioimmunoblastic	Mixed	No	Steroids, intravenousImmunoglobulin, chemotherapy
Fozza et al. 2009 [29]	Marginal zone	Warm	Yes	Steroids

Abbreviations: CHOP (cyclophosphamide, hydroxydaunorubicin, vincristine, prednisone/prednisolone); CVP (cyclophosphamide, vincristine, prednisone/prednisolone); NHL (non-Hodgkin lymphoma).
